# Are behaviour change techniques and intervention features associated with effectiveness of digital cardiac rehabilitation programmes? A systematic review protocol

**DOI:** 10.12688/hrbopenres.13355.1

**Published:** 2021-08-11

**Authors:** Eanna Kenny, John W. McEvoy, Jenny McSharry, Linda M. Collins, Rod S. Taylor, Molly Byrne

**Affiliations:** 1 Health Behaviour Change Research Group, School of Psychology, National University of Ireland Galway, Galway, Ireland; 2 National Institute for Prevention and Cardiovascular Health, School of Medicine, National University of Ireland Galway, Galway, Ireland; 3 Departments of Social and Behavioral Sciences and Biostatistics, School of Global Public Health, New York University, New York, NY, USA; 4 MRC/CSO Social and Public Health Sciences Unit & Robertson Centre for Biostatistics, Institute of Health and Well Being, University of Glasgow, Glasgow, UK

**Keywords:** Cardiovascular disease, cardiac rehabilitation, digital, behaviour change, systematic review

## Abstract

**Background: **Cardiovascular disease (CVD) is the leading cause of death worldwide. Cardiac rehabilitation (CR) is a complex intervention that aims to stabilise, slow, or reverse the progression of CVD and improve patients’ functional status and quality of life. Digitally delivered CR has been shown to be effective and can overcome many of the access barriers associated with traditional centre-based delivered CR programmes. However, there is a limited understanding of the behaviour change techniques (BCTs) and intervention features that maximise the effectiveness of digital programmes. Therefore, this systematic review will aim to identify the BCTs that have been used in digital CR programmes and to determine which BCTs and intervention features are associated with programme effectiveness.

**Methods: **PubMed, MEDLINE (Ovid), EMBASE, CINHAL, PsycINFO and Cochrane Central Register of Controlled Trials will be searched from inception to June 2021 for randomised controlled trials of digital CR with CVD patients. Screening, data extraction, intervention coding and risk of bias will be performed by one reviewer with a second reviewer independently verifying a random 20% of the articles. Intervention content will be coded using the behaviour change technique taxonomy v1 and the Template for Intervention Description and Replication (TIDieR) checklist and intervention features will be identified. A meta-analysis will be conducted to calculate the pooled effect size of each outcome, and meta-regression analyses will investigate whether intervention features and the presence and absence of individual BCTs in interventions are associated with intervention effectiveness.

**Discussion: **The review will identify BCTs and intervention features that are associated with digital CR programmes and adopt a systematic approach to describe the content of these programmes using the BCT taxonomy (v1) and TIDieR checklist. The results will provide key insights into the content and design of successful digital CR programmes, providing a foundation for further development, testing and refinement.

## Introduction

Cardiovascular disease (CVD) is the number one cause of death worldwide, accounting for almost a third (31%) of global deaths ^1^. Cardiac rehabilitation (CR) is a complex intervention that aims to stabilise, slow, or reverse the progression of CVD and improve participant functional status and quality of life. Systematic reviews of CR have shown it is effective at improving lifestyle behaviours and quality of life and can achieve significant reductions in morbidity, mortality and hospital admissions among people with CVD ^2^. Despite these benefits, CR is underutilised with participation rates in Europe as low as 36% and dropout between 12-56% ^3^. A range of barriers to uptake have been identified, at both the service and system level (e.g., poor referral system), and patient level (e.g., transport, cost, perceptions of CVD and CR) ^4^. Furthermore, service delivery has been severely impacted by unit closures and staff redeployment due to the coronavirus disease 2019 (COVID-19) pandemic ^5– 7^, generating an increased need for alternative methods of delivering CR.

CR is a multifaceted intervention and the ‘active ingredients’ of the intervention are still unclear ^8^. This is possibly due to the variety of components and techniques often included in CR, which make it difficult to tease apart the effectiveness of individual parts of the intervention. Digital health interventions (DHIs) have potential as scalable tools to improve health and healthcare delivery. They also allow easier experimental manipulation of intervention components for the purpose of understanding how exactly interventions work and what components of the intervention work better than others ^9^. Previous systematic reviews have indicated that secondary prevention DHIs are effective at improving outcomes such as CVD events, hospitalisations, and all-cause mortality ^10^, as well as modifiable risk factors including physical activity and medication adherence ^11^. Furthermore, a systematic review of eHealth cardiac rehabilitation found a positive impact on physical activity, daily steps, quality of life and rehospitalization ^12^. Digitally delivered CR has been shown to be at least equally as effective as traditional centre-based CR ^13– 15^ and can address many of the barriers associated with attending centre-based CR ^4^. The potential for digital CR is significant, however, a greater understanding of what constitutes an effective digital CR programme is necessary to maximise efficiency and scalability ^16^. 

Developments in behavioural science have provided tools to support the clear specification of intervention content. An example of this is the behaviour change technique (BCT) taxonomy (v1) ^17^, a comprehensive list of 93 BCTs which allows the ‘active ingredients’ of interventions to be systematically described and replicated. A further example is the Template for Intervention Description and Replication (TIDieR) checklist ^18^ which details the why, what, who, where, and how of intervention delivery. More recently, the mode of delivery ontology v1 ^19^ has been developed as a tool for describing the mode of delivery of behaviour change interventions in a consistent and coherent manner. These tools have the potential to enable a greater understanding of the content of effective digital CR programmes, while also improving evidence comparison and study replication. To our knowledge, no systematic review has evaluated digital CR programmes using these tools.

### Objectives

Therefore, the aim of this systematic review is to answer the following two research questions:

1. What BCTs have been used in digital CR programmes?
2. Which BCTs and intervention features, including mode of delivery, theoretical framework, dose, intensity, and frequency, are associated with digital CR programme effectiveness?

## Methods

This protocol has been reported using the Preferred Reporting Items for Systematic Reviews and Meta-Analyses Protocols (PRISMA-P) guidelines ^20^.

### Eligibility criteria

Eligible studies will be peer-reviewed publications in English, that include adults (>18 years old) with any form of heart disease (coronary heart disease, acute coronary syndrome, congenital heart disease, heart failure, valvular heart disease). Studies will be included if they use an randomised control trial (RCT) design to assess the effectiveness of a digital CR programme when compared to usual care. The intervention must be delivered at least in part via the internet or a smartphone application; interventions that solely use landline telephone communication will be excluded. Studies will be included if they report any behavioural outcomes (physical activity, diet, smoking, alcohol use, medication adherence) as either the primary or secondary outcome.

### Information sources

A comprehensive database search will be conducted using PubMed, MEDLINE (Ovid), EMBASE, CINHAL, Ovid PsycINFO and Cochrane Central Register of Controlled Trials. Databases will be searched from inception to present date. Included publications will be forward and backward reference searched to identify additional relevant studies. Study authors will be contacted where information is missing and/or the full text article is unavailable.

### Search strategy

The search strategy will be developed based upon previous systematic reviews ^10– 12^ and in consultation with a specialist librarian. It will include a combination of medical subject headings (or equivalent) and free text terms. Table 1 provides an example of the search strategy for MEDLINE (Ovid); the search will be adapted for each database.

**Table 1.** Search strategy for MEDLINE (Ovid).

Search no.	Search terms
1.	Exp cardiovascular diseases/
2.	Exp cardiology/
3.	(coronary adj3 (artery OR disease)).ti,ab.
4.	(myocardial adj3 (isch?em* OR infarct*)).ti,ab.
5.	(CHD OR CVD).ti,ab.
6.	(cardiac OR cardiovascular).ti,ab.
7.	OR/1-6
8.	Exp cell phone/
9.	(cellphone OR cell phone OR mobile phone OR cellular phone OR smartphone OR smart phone).ti,ab.
10.	text messag*.ti,ab.
11.	Exp telemedicine/
12.	(telehealth OR tele health OR telemedicine OR tele medicine OR telerehab* OR tele rehab*). ti,ab.
13.	Exp internet/
14.	(web OR internet OR online).ti,ab.
15.	(digital health OR tech OR virtual).ti,ab.
16.	(ehealth OR e health OR mhealth OR m health).ti,ab.
17.	OR/8-16
18.	Exp exercise/
19.	(exercis* OR physical activ* OR diet OR nutrition OR tobacco OR smoking OR adherence).ti,ab.
20.	OR/18-19
21.	Exp rehabilitation/
22.	(interven* OR program* OR treatment OR (cardiac adj3 rehabilitation)).ti,ab.
23.	OR/21-22
24.	7 AND 17 AND 20 AND 23

### Data management

The results from all database searches will be imported into Endnote X20. Duplicates will be removed by the software and then checked manually by the main reviewer (EK). Articles will then be exported to Rayyan ^21^ for screening.

### Selection process

Articles will be screened by abstract and full text by one reviewer (EK), a second reviewer will independently screen a random 20% of the articles. Any disagreements will be resolved through discussion and the consultation of a third reviewer. Reasons for inclusion/exclusion will be recorded and a Preferred Reporting Items for Systematic Reviews and Meta-Analyses (PRISMA) flow diagram will be completed ^22^.

### Data extraction

One reviewer (EK) will extract data from the included studies using a pre-piloted data extraction form, with a random 20% checked for accuracy by a second independent reviewer. The following data will be extracted:

- General: author(s), year, and country of origin;
- Study characteristics: aims/objectives of the study, study design, inclusion/exclusion criteria, recruitment methods, sample size, and unit of allocation;
- Participants: age, sex, setting, diagnosis, and baseline characteristics;
- Intervention: mode of delivery using the mode of delivery ontology v1 ^19^, duration, BCTs based on the BCT taxonomy (v1) ^17^, quality of intervention reporting using the TIDieR checklist ^18^, CR components based on the core components of home-based CR programmes outlined in Thomas *et al*. ^23^, and theoretical basis;
- Outcomes: Primary outcomes, and secondary outcomes.

Where a study is described across multiple publications, an attempt will be made to extract and combine all the available data. Study authors will be contacted if data is missing. 

### Outcomes

The primary outcomes are changes in health-related behaviours (physical activity, diet, smoking, alcohol, sedentary behaviour and medication adherence) as CR is an intervention aimed primarily at improving modifiable CVD risk factors. Additional outcomes will include psychological well-being, quality of life, adherence rates, lipid profile, blood pressure, weight/body mass index (BMI), cardiac events, revascularisation, rehospitalisation and mortality. 

### Risk of bias in individual studies

Study quality will be assessed using the Cochrane risk of bias yool (version 2) ^24^. This tool assesses study quality on the domains of selection bias, performance bias, detection bias, attrition bias, reporting bias and other biases. One reviewer (EK) will complete the assessment and a second reviewer will check a random 20% of the studies.

### Data synthesis

A summary of the information extracted from each study, including outcomes reported, BCTs and content from the items on the TIDieR checklist, will be provided in narrative and tabular form. A meta-analysis to calculate the pooled effect size of each outcome will be considered if there is sufficient homogeneity of outcomes across studies, with the Higgins I ^2^ statistic being used to assess heterogeneity. A random-effect or fixed-effect model will be chosen depending on the level of heterogeneity of intervention effects. The meta-analysis will be conducted using Review Manager (RevMan) version 5.4 ^25^. For continuous variables, the mean difference will be calculated if the same measurement scale was used, alternatively the standardised mean difference (SMD) will be calculated (with 95% CI [confidence interval]). For dichotomous variables, proportions will be compared using risk ratios (with 95% CIs). If a meta-analysis is not possible, a narrative synthesis will be conducted.

A meta-regression analysis will be performed if there are at least 6-10 studies for a continuous study-level variable ^26^. The meta-regression analysis will examine whether intervention features (e.g., mode of delivery, theoretical framework, dose, intensity, and frequency) and the presence and absence of individual BCTs in the interventions are associated with each outcome.

Subgroup analyses will be performed where appropriate and possible, comparing studies based on intervention components, duration of the intervention and type of control used (centre-based versus home-based CR).

### Meta-bias(es)

 Study protocols will be assessed for evidence of selective reporting within studies. Reporting bias will be analysed using funnel plots.

### Confidence in cumulative evidence

The quality of evidence will be assessed using the Grading of Recommendations Assessment, Development and Evaluation guidelines (GRADE) ^27^.

## Discussion

Digitally delivered CR has the potential to improve behavioural outcomes for patients with CVD and to overcome some of the barriers associated with traditional CR service delivery. This review will identify the BCTs and intervention features that are associated with effective digital CR programmes and adopt a systematic approach to describe the content of these programmes using the BCT taxonomy (v1) and TIDieR checklist. This detailed intervention description will provide insight into the content, design and active ingredients of successful digital CR programmes, providing a foundation for further development, testing and refinement. This systematic review is being conducted in the broader context of developing a conceptual model of digital CR as the first step in an optimisation trial. 

## Data availability

No data are associated with this article.
