# Supplementary material for: Are behaviour change techniques and intervention features associated with effectiveness of digital cardiac rehabilitation programmes? A systematic review protocol
Source: HRB Open Res. 2021 Aug 11;4:88. [Version 1] doi: 10.12688/hrbopenres.13355.1 (PMC8767424; doi:10.12688/hrbopenres.13355.1)
Supplement: Peer review history

# Peer review: Reviewer response for version 1

**DOI:** 10.21956/hrbopenres.14543.r31218

**Published:** 2022-01-18

**Authors:** Garyfallia Pepera, Varsamo Antoniou, Ladislav Batalik

**Affiliations:** 1 Physiotherapy Department, Faculty of Health Sciences, University of Thessaly, Thessaly, Greece; 2 Department of Rehabilitation, University Hospital of Brno, Brno, Czech Republic

We read with interest the resent systematic protocol by Kenny *et al. *aiming to the identification of the behaviour change techniques (BCTs) that are used in digital cardiac rehabilitation (CR) programs and the determination of the ones that are mostly correlated to the enhancement of the CR program effectiveness (Sandercock *et al.* (2013 ^1^)).

 We strongly agree to the authors’ assertion that the several barriers related to the underutilization of the traditional center-based CR programs can be addressed by the implementation of digital health interventions (DHIs) (Winnige et al. (2021 ^2^)) Indeed, a recent critical review of ours has proclaimed the potential effective role of telehealth interventions as alternative sufficient methods of delivering CR during over demanding socioeconomic conditions, such is the ongoing COVID-19 pandemic (Stefanakis *et al. *(2021 ^3^), Pepera *et al. *(2022 ^5^))

 Though,  tele-delivered CR is indeed a complex multi-disciplinary intervention, that intergrades several different components and techniques in its design that are aiming to the improvement of  varied aspects of the cardiac patients’(CVD) health status. Thus, we also consent to the need to specify and qualify the content of the CR program components and their contribution, separately or combined, in the maximization of the DHIs efficacy and efficiency.

 We feel that this ongoing systematic review could provide the necessary background to a more compete design of the CR telehealth interventions and a more profound understanding of the theoretical framework and the practical features (dose, intensity, frequency) of the BCTs used in digital CR programs (Batalik *et al. *(2021 ^4^))

 The use of reliable methods for specifying, interpreting and implementing the active ingredients of interventions to change behaviors by this systematic review, such are the behavior change technique (BCT) taxonomy (v1) and the Template for Intervention Description and Replication (TIDieR) checklist could lead to a reliable identification and categorization of the most effective digital CR features; thus offering CR researchers and specialists a useful tool for a more sufficient, aim-specific orientated digital CR program implementation.

 Baring in mind that DHIs aim to act as secondary preventive interventions that could modify and improve several cardiac risk factors, it is important to gain knowledge over the different characteristics and features of the multiple tools that are being intergraded in their design and better comprehend the way these tools can lead to behavioral changes within the targeted cardiac population.

 Nowadays, more than ever, there is an urgent need to adopt a more well-orientated approach to CR design and implementation, based on the choice of the most appropriate CR components that could lead to an effective CR implementation.

Is the study design appropriate for the research question?

Yes

Is the rationale for, and objectives of, the study clearly described?

Yes

Are sufficient details of the methods provided to allow replication by others?

Yes

Are the datasets clearly presented in a useable and accessible format?

Yes

Reviewer Expertise:

Cardiac Rehabilitation

We confirm that we have read this submission and believe that we have an appropriate level of expertise to confirm that it is of an acceptable scientific standard.

## References

[1] : Cardiorespiratory fitness changes in patients receiving comprehensive outpatient cardiac rehabilitation in the UK: a multicentre study. *Heart* .2013;99(11) : 10.1136/heartjnl-2012-303055 785-90 10.1136/heartjnl-2012-303055 23178183

[2] : Validity and Reliability of the Cardiac Rehabilitation Barriers Scale in the Czech Republic (CRBS-CZE): Determination of Key Barriers in East-Central Europe. *International Journal of Environmental Research and Public Health* .2021;18(24) : 10.3390/ijerph182413113 10.3390/ijerph182413113 PMC870171534948722

[3] : Exercise-based cardiac rehabilitation programs in the era of COVID-19: a critical review. *Reviews in Cardiovascular Medicine* .2021;22(4) : 10.31083/j.rcm2204123 10.31083/j.rcm2204123 34957758

[4] : Is the Training Intensity in Phase Two Cardiovascular Rehabilitation Different in Telehealth versus Outpatient Rehabilitation?. *J Clin Med* .2021;10(18) : 10.3390/jcm10184069 10.3390/jcm10184069 34575185PMC8466823

[5] : Epidemiology, risk factors and prognosis of cardiovascular disease in the Coronavirus Disease 2019 (COVID-19) pandemic era: a systematic review. *IMR Press* .2022;23(1) : 10.31083/j.rcm2301028 10.31083/j.rcm2301028 35092220

# Peer review: Reviewer response for version 1

**DOI:** 10.21956/hrbopenres.14543.r30603

**Published:** 2021-11-15

**Authors:** Jonathan C Rawstorn

**Affiliations:** 1 Institute for Physical Activity and Nutrition, Deakin University, Geelong, VIC, Australia

Thank you for the opportunity to review this protocol for a systematic review that will aim to synthesise current evidence about digital cardiac rehabilitation including the integration of behaviour change techniques, intervention features, effects on health behaviours and modifiable CVD risk factors, and potential impact of behaviour change techniques and intervention features on outcome effects.

 The rationale for this review is solid and the results will likely be impactful for many researchers and clinicians working in the field of digital cardiac rehabilitation. This protocol clearly and succinctly describes relevant aspects of the study design and methods. Terminology suggests the search strategy may not yet have been finalised; however, the provided example looks appropriate for the study aims and, and given the final search strategy will likely be published together with the results in due course, I don't see this as a major problem.

 I only have two minor comments:

1. It is unclear whether the authors have registered the review in the PROSPERO database. If so, the registration number could be included in this submission.
2. Articulating the status or current progress of the review would help to determine whether the manuscript presents a prospective or retrospective account of the design and methods.

I look forward to reading the results of this review in due course.

Is the study design appropriate for the research question?

Yes

Is the rationale for, and objectives of, the study clearly described?

Yes

Are sufficient details of the methods provided to allow replication by others?

Yes

Are the datasets clearly presented in a useable and accessible format?

Not applicable

Reviewer Expertise:

cardiac rehabilitation, digital health, systematic reviews

I confirm that I have read this submission and believe that I have an appropriate level of expertise to confirm that it is of an acceptable scientific standard.
